# Aging related cognitive changes associated with Alzheimer's disease in Down syndrome

**DOI:** 10.1002/acn3.571

**Published:** 2018-05-20

**Authors:** Nicholas C. Firth, Carla M. Startin, Rosalyn Hithersay, Sarah Hamburg, Peter A. Wijeratne, Kin Y. Mok, John Hardy, Daniel C. Alexander, The LonDownS Consortium, André Strydom

**Affiliations:** ^1^ Centre for Medical Image Computing Department of Computer Science UCL London WC1E 6BT United Kingdom; ^2^ Department of Forensic and Neurodevelopmental Sciences Institute of Psychiatry, Psychology & Neuroscience Kings College London London SE5 8AF United Kingdom; ^3^ Division of Psychiatry UCL London WC1E 6BT United Kingdom; ^4^ LonDownS Consortium London United Kingdom; ^5^ Department of Molecular Neuroscience Institute of Neurology UCL London WC1N 3BG United Kingdom; ^6^ Division of Life Science Hong Kong University of Science and Technology Hong Kong SAR China; ^7^ Reta Lila Weston Institute Institute of Neurology UCL London WC1N 3BG United Kingdom; ^8^ South London and Maudsley NHS Foundation Trust Bethlem Royal Hospital Monks Orchard Road Beckenham Kent BR3 3BX United Kingdom

## Abstract

**Objective:**

Individuals with Down syndrome (DS) have an extremely high genetic risk for Alzheimer's disease (AD), however, the course of cognitive decline associated with progression to dementia is ill‐defined. Data‐driven methods can estimate long‐term trends from cross‐sectional data while adjusting for variability in baseline ability, which complicates dementia assessment in those with DS.

**Methods:**

We applied an event‐based model to cognitive test data and informant‐rated questionnaire data from 283 adults with DS (the largest study of cognitive functioning in DS to date) to estimate the sequence of cognitive decline and individuals’ disease stage.

**Results:**

Decline in tests of memory, sustained attention/motor coordination, and verbal fluency occurred early, demonstrating that AD in DS follows a similar pattern of change to other forms of AD. Later decline was found for informant measures. Using the resulting staging model, we showed that adults with a clinical diagnosis of dementia and those with *APOE* 3:4 or 4:4 genotype were significantly more likely to be staged later, suggesting that the model is valid.

**Interpretation:**

Our results identify tests of memory and sustained attention may be particularly useful measures to track decline in the preclinical/prodromal stages of AD in DS whereas informant‐measures may be useful in later stages (i.e. during conversion into dementia, or postdiagnosis). These results have implications for the selection of outcome measures of treatment trials to delay or prevent cognitive decline due to AD in DS. As clinical diagnoses are generally made late into AD progression, early assessment is essential.

## Introduction

Down syndrome (DS) is due to full or partial trisomy, translocation, or mosaicism of chromosome 21, and is associated with intellectual disability (ID).^1^ DS is also a genetic cause of Alzheimer's disease (AD), largely due to triplication of the amyloid precursor protein (*APP*) gene at 21q21.3.^2^, ^3^, ^4^ AD neuropathology is universally present in adults with DS from their fourth decade,^5^, ^6^ driving an elevated risk for dementia due to AD that is estimated to reach 80% by age 65,^7^ though the age of clinical dementia onset shows large variability.^8^ The age of onset for dementia in DS is similar to that in familial AD due to mutations in the known AD causing genes ‐ *APP*, presenilin 1 (*PSEN1*) and presenilin 2 (*PSEN2*),^9^ with mean age of diagnosis around 55, and an interquartile range of approximately 50–59 years of age.^8^ However, unlike familial AD, the sequence and course of dementia in DS is less well‐described, despite this population currently accounting for the majority of genetic AD cases.

In individuals with DS, the development of dementia needs to be understood in the context of a complex cognitive phenotype that not only includes general ID, but also specific impairments in executive function, memory, language, and motor domains.^10^, ^11^ Such pre‐existing impairments in those with DS need to be distinguished from subsequent decline, and in combination with varying baseline abilities and limitations in speech abilities can make the interpretation of cognitive test data, and thus clinical diagnosis, difficult.^12^, ^13^

The evaluation of longitudinal change suggestive of AD in DS, within and across different cognitive domains, poses significant challenges. In addition to the aforementioned difficulties of assessing decline in the presence of varying degrees of premorbid ID, it is not trivial to understand the long‐term longitudinal progression of a disease when the majority of studies sample populations at different stages of disease progression, by taking cross‐sectional or short‐term longitudinal measurements.^14^ Data‐driven methods have become a valuable tool for studying long‐term disease progression due to their ability to estimate long‐term trends from cross‐sectional and short‐term longitudinal snapshots of cohorts, and can be adjusted for variability in baseline ability. The event‐based model (EBM) is one such method capable of estimating orderings of multimodal measurements and staging participants.^15^ The EBM has been applied previously to neuroimaging, cerebrospinal fluid (CSF), and cognitive markers in sporadic AD,^16^ and more recently was reformulated to model more complex cognitive datasets in young onset AD and posterior cortical atrophy.^17^

The aim of this work was to characterize the cognitive deterioration associated with the development of AD in DS. We applied the data‐driven EBM to markers of cognitive and informant‐rated ability of individuals with DS to estimate the order of cognitive decline and assign participants to a disease stage. We further aimed to determine the effect of a clinical diagnosis of dementia and *APOE* genotype on stage, given that *APOE* genotype is strongly associated with age of onset of dementia due to AD, with the *e4* allele driving earlier onset and increased risk and the *e2* allele reducing risk.^18^

## Methods

### Ethics and consent

We obtained ethical approval from the National Health Service Research Ethics Committee for the LonDownS consortium's longitudinal study of cognitive ability in DS, including approval for collection of DNA samples (13/WA/0194). Individuals with capacity to consent for themselves provided written informed consent, and for those who did not have decision‐making capacity, a consultee was approached to indicate their agreement to the individual's participation, in accordance with the UK Mental Capacity Act 2005.

### Participants

We recruited individuals with a clinical diagnosis of DS aged 16 years and older living in family homes and residential facilities across England and Wales via a volunteer database, support groups, and local National Health Service (NHS) Trust sites. Participants with any acute physical or mental health condition were excluded from participation until they had recovered.

Due to the increased risk of people with DS developing AD neuropathology characterized by amyloid deposition beyond age 35 as demonstrated by neuropathological and amyloid positron emission tomography studies,^2^, ^19^, ^20^ we used age 35 to split participants into two age groups. The young adult (YA) group, aged 16–35, were likely performing at or near to their cognitive peak, while the older adult (OA) group, aged 36 and older, were expected to have AD neuropathology with individuals presenting with varying degree of cognitive decline. We therefore defined the YA group as a predecline “control” group, while the OA group was used as a “pathological” group in the model due to the likelihood that many of the OA group have already undergone cognitive deterioration, whether they have been diagnosed with dementia or not. This approach was chosen because we wished to identify the stages of cognitive decline associated with prodromal AD in DS leading up to clinical dementia diagnosis. In subsequent analyses, we used dementia status to test the validity of the model. The semisupervised mixture modeling technique used (Event‐based model) has been previously shown to be capable of discerning splits from heterogeneous inputs, such as the YA and OA groups in this work.

### Cognitive test battery

We selected tests and outcomes from the LonDownS cognitive battery that showed good psychometric properties across the age groups:

1. General cognitive abilities were assessed using raw scores from the verbal and nonverbal subscales of the Kaufman Brief Intelligence Test 2 (KBIT‐2).^21^
2. Visuospatial associate memory was assessed with the first trial memory score from the CANTAB paired associates learning (PAL) task.^22^ This sums the number of pattern positions correctly recalled after their first presentation in all stages attempted.
3. Object memory was assessed using an adapted form of the Fuld object memory test.^23^ This task provides measures of immediate and 5‐min delayed memory recall.
4. Orientation abilities were assessed by asking participants questions about when it was (the day, month, and year), and where they were.^24^
5. The intra/extra dimensional set shift (IED) task is a measure of rule learning and set shifting from the CANTAB.^22^ Here, we used the total number of stages completed.
6. An adapted version of the Tower of London for individuals with an ID assessed working memory and planning.^11^, ^25^
7. To measure semantic verbal fluency participants were asked to name as many animals as possible in 1 min.
8. The mean latency of responses in the simple reaction time (SRT) task from the CANTAB was used as a measure of attention and motor abilities.^22^, ^26^
9. The finger‐nose pointing test is a clinical measure of motor coordination.^27^
10. The car and motorbike score from the NEPSY‐II – visuomotor precision task assesses hand‐eye coordination.^28^

Informants (relatives or paid carers) completed standardized questionnaires. These included:

1. The Short Adaptive Behavior Scale (short ABS),^29^ adapted from the Adaptive Behavior Scale – Residential and Community (Part I),^30^ records participants’ everyday adaptive abilities.
2. The Dementia Questionnaire for People with Learning Disabilities (DLD) measures behaviors associated with cognitive decline in people with ID over the last two months.^31^ Cognitive and social domains scores were included.
3. The Observer Memory Questionnaire (OMQ) measures individuals’ memory abilities over the last 2 months

We developed a revised, shorter version, by selecting the most reliable items appropriate for use in adults with ID.^32^

Further information about the LonDownS participants, cognitive assessments, and informant questionnaires can be found in (Startin et al. 2016^11^), with a summary of tests and outcomes used in Table 1.

**Table 1:** Summary of assessments used

	Test name	Primary abilities assessed	Description	Outcomes and score ranges
Participant tests	Kaufmann brief intelligence test 2 (KBIT‐2)	General cognitive abilities	Subtests assess participants’ verbal abilities (verbal knowledge and riddles) and nonverbal abilities (matrices)	Verbal raw score (0–108); Nonverbal raw score (0–46)
	CANTAB paired associates learning (PAL)	Visuospatial associate memory	Participants were required to remember locations of an increasing number of patterns, hidden behind boxes on a computer screen	First trial memory score (0–26)
	Object memory test	Recall memory	Participants were required to name and remember a series of objects, then recall them in two immediate trials and one 5 min delayed trial	Immediate recall (0–14); Delayed recall (0–7)
	CAMCOG orientation	Orientation	Assesses participants’ knowledge of when it is and where they are	Total score (0–12)
	CANTAB intra/extra dimensional set shift (IED)	Rule learning and set shifting	Participants were required to learn rules about which was the ‘correct’ of two presented patterns on a computer screen, with a rule change after six consecutive correct trials	Number of stages completed (0–9)
	Tower of London	Working memory and planning	Participants were required to move beads on a board to match presented configurations.	Total score (0–10)
	Verbal fluency	Semantic verbal fluency	Participants were asked to name as many animals as possible in 1 min	Number of unique animals (0‐N/A)
	CANTAB simple reaction time (SRT)	Attention/motor abilities	Participants were required to press a button as soon as a white square appeared on a computer screen	Mean latency (N/A)
	Finger‐nose pointing	Motor coordination	Participants alternatively touch their nose and a red circle 45 cm away for 20 sec	Total number of times the circle is touched (0‐N/A)
	NEPSY‐II visuomotor precision	Hand‐eye coordination	Participants were timed as they traced around train, car, and motorbike tracks, with times and number of errors for each track used to determine overall scores	Car and motorbike score (0–52)
Informant ratings	Short adaptive behavior Scale (Short ABS)	Adaptive abilities	Informants answer questions about everyday adaptive abilities	Total score (0–113)
	Dementia questionnaire for people with learning disabilities (DLD)	Memory and orientation/adaptive abilities	Informants answer questions about behaviors associated with cognitive decline over the last 2 months	Cognitive abilities (0–44); Social abilities (0–60)
	Revised observer memory questionnaire (OMQ)	Memory	Informants answer questions about individuals’ memory abilities over the last 2 months	Total score (18–90)

### Imputation

Floor effects and difficulty to engage in cognitive tasks are a significant issue in studies of cognitive decline in individuals with DS, and excluding those who score at floor or who do not engage could significantly bias analyses. We therefore imputed scores as follows: individuals who attempted tasks but were clearly unable to understand task instructions were allocated a score of zero for outcomes aside from SRT mean latency, where the poorest score recorded was given. Missing items from the DLD and OMQ were imputed for up to 15% of items within each domain with the nearest integer to the mean value of completed scores.

### ID severity score

Premorbid ID level was defined according to the ICD10 diagnostic system, and classified into three levels based on caregiver's reports of the individual's best ever level of functioning ‐ mild, moderate, and severe ID, corresponding to the general functional abilities associated with IQ levels of 50–69, 35–49, and <35, respectively, as described elsewhere.^33^

### Dementia diagnoses

Dementia diagnoses were used to test the validity of the staging model, and were defined as the presence of an existing, independent clinical diagnosis from each individual's clinician after comprehensive clinical assessment. Clinical diagnosis has previously been shown to be reliable.^34^ None of the tests used in the EBM were used to inform diagnoses.

### Genetic analysis

Participants’ DS status was confirmed genetically using saliva or blood samples where possible; following DNA extraction, genome‐wide single nucleotide polymorphism (SNP) genotyping was performed using an Illumina OmniExpressExome array (San Diego, CA) at UCL Genomics, then assembled and visually inspected in GenomeStudio to confirm the presence of an additional copy of chromosome 21, mosaicism, or translocation. *APOE* genotype was determined using a Thermo Fisher Scientific Taqman assay for SNPs rs7412 and rs429358 (Waltham, MA).

### Event‐based model

Scores from the cognitive tests and informant questionnaires, controlled for ID level, were used as input in the EBM, with scores termed as ‘biomarkers’ for the remainder of this paper, for brevity and to be consistent with previous descriptions of the model.^15^, ^16^ Unimodal, two‐component, nonparametric mixture models were fit for each of the biomarkers, these models were then used to assign probabilities $P(x|E_{i})$ and $P(x|\neg E_{i})$ of a biomarker measurement, *x*, being abnormal (event *E*_*i*_ has occurred) or normal (*E*_*i*_ has not occurred) that is the measurement indicating dementia or not respectively.^17^ The OA and YA groups were used to define the initial components in each mixture model, by defining each group as a component and then fitting kernel density estimations to each group separately. The fitting procedure then uses these initial components to estimate two components corresponding to a dementia and nondementia subpopulation. This is possible as mixture modeling is a semisupervised method capable of learning underlying patterns in the data that correspond to dementia or not, despite being only provided with the YA and OA labels. The EBM was then used to estimate the maximum likelihood ordering of events. In this work the ordering of events corresponds to the order of decline on cognitive tests and informant questionnaires, which transition outside of a premorbid range due to cognitive decline associated with AD progression. The EBM was used as previously described,^35^ briefly an event sequence S, was optimized using Markov chain Monte Carlo (MCMC) sampling to maximize the probability of the full set of data, *X* (all biomarkers from all individuals), given by

(1) $$P\left(X|S\right)=\prod_{i=1}^{J}\left[\sum_{k=0}^{I}\left(\prod_{i=1}^{k}P\left(x_{\mathrm{ij}}|E_{i}\right)\prod_{i=k+1}^{I}P\left(x_{\mathrm{ij}}|\neg E_{i}\right)\right)\right]$$

where i∈I is the biomarker index and j∈J is the participant number. The fitting procedure identifies the maximum likelihood sequence $\hat{S}$, from which disease stages were estimated for individuals given their test scores. Similar to previous descriptions of the EBM (e.g.^16^), we used the stage, $k_{j}$, which has the highest probability given the data and our sequence, i.e.

$${\hat{k}}_{j}=\text{argma}x_{k}P\left(X_{i}|\hat{S},k\right)=\text{argma}x_{k}\prod_{i=1}^{k}P\left(x_{\mathrm{ij}}|E_{i}\right)\prod_{i=k+1}^{I}P\left(x_{\mathrm{ij}}|\neg E_{i}\right)$$

Disease stages for adults in the OA and YA groups were then compared, as were stages of adults in the OA group with and without a clinical diagnosis of dementia, and stages for individuals with different *APOE* genotypes for the YA and OA groups separately. The APOE4 group was defined as those possessing a copy of the *APOE* e4 allele (*APOE* 3:4 and 4:4), while the APOE2 group included individuals possessing a copy of the *APOE* e2 allele (*APOE* 2:2 and 2:3), and the APOE3 group consisted of those possessing two copies of the *APOE* e3 allele (*APOE* 3:3). Individuals with *APOE* 2:4 genotype were omitted from this analysis. All group comparisons used Mann–Whitney *U* tests.

## Results

This analysis included 283 participants, with details of participants included in the study summarized in Table 2.

**Table 2:** Demographic data and mean (SD) neuropsychological scores for younger adult and older adult groups

		Younger adults	Older adults
Participants	*n*	119	164
	DS status confirmed	*n* = 115	*n* = 156
		110 trisomy	150 trisomy
		1 translocation	1 translocation
		3 mosaic	5 mosaic
		1 partial trisomy	
	Age	25.24 (5.58)	49.58 (7.47)***
	Gender	57 M; 62 F	87 M; 77 F
	LD level	45 mild; 63 moderate; 11 severe	69 mild; 67 moderate; 28 severe
	Clinical dementia status	119 no dementia	121 no dementia; 43 dementia
	*APOE* genotype	*n* = 111	*n* = 151
		*n* = 19 *APOE* 2:2 or 2:3	*n* = 22 *APOE* 2:2 or 2:3
		*n* = 60 *APOE* 3:3	*n* = 90 *APOE* 3:3
		*n* = 31 *APOE* 3:4 or 4:4	*n* = 35 *APOE* 3:4 or 4:4
		*n* = 1 *APOE* 2:4	*n* = 4 *APOE* 2:4
Tests	KBIT‐2 raw verbal	*n* = 119; 34.95 (16.99)	*n* = 164; 23.02 (18.77)***
	KBIT‐2 raw non‐verbal	*n* = 119; 15.00 (6.94)	*n* = 154; 9.78 (7.43)***
	PAL first trial memory	*n* = 107; 10.19 (5.67)	*n* = 144; 4.53 (5.61)***
	IED stages complete	*n* = 108; 6.52 (2.59)	*n* = 144; 3.41 (3.06)***
	SRT mean latency	*n* = 104; 695.72 (455.97)	*n* = 137; 1423.08 (793.61)***
	Delayed object memory	*n* = 109; 5.78 (1.52)	*n* = 151; 3.60 (2.66)***
	Immediate object memory	*n* = 109; 10.26 (3.00)	*n* = 151; 6.41 (4.75)***
	NEPSY car motorbike score	*n* = 116; 17.12 (9.56)	*n* = 151; 8.05 (8.91)***
	Tower of London	*n* = 113; 7.24 (3.12)	*n* = 155; 4.41 (3.97)***
	Finger nose	*n* = 114; 11.02 (5.25)	*n* = 155; 6.09 (5.48)***
	Verbal fluency	*n* = 115; 10.81 (5.91)	*n* = 157; 6.10 (5.92)***
	Orientation	*n* = 113; 9.55 (3.56)	*n* = 155; 6.52 (4.71)***
	DLD cognitive	*n* = 111; 7.57 (8.35)	*n* = 140; 15.68 (12.75)***
	DLD social	*n* = 114; 9.31 (6.87)	*n* = 143; 14.66 (10.52)***
	Short ABS total	*n* = 114; 79.60 (19.46)	*n* = 144; 64.79 (27.00)***
	OMQ revised	*n* = 116; 43.42 (12.31)	*n* = 141; 56.38 (17.16)***

Significant differences between groups have been highlighted, *p *<* *0.005 (***).

### Event sequences

**Figure 1:**
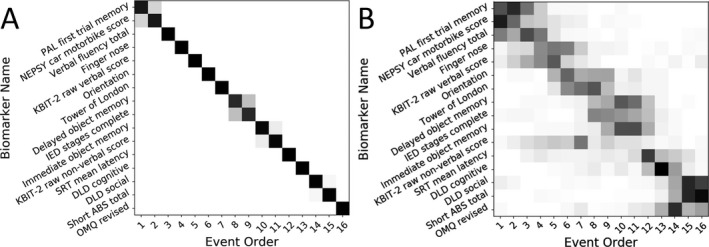
Positional variance diagrams show the maximum likelihood event sequence. Each entry represents the proportion of samples with each biomarker in each position ranging from 0 in white to 1 in black. (A) Positional variance diagram of the Markov chain Monte Carlo samples generated during fitting of the event‐based model (B) diagram of the samples generated during bootstrapping of the model.

To account for the severity of cognitive deficits not caused by dementia development but instead due to the intellectual impairments associated with DS, ID level was used to estimate residuals in the YA group for each biomarker using linear regression coefficients, then these coefficients were used to calculate residuals for all individuals’ biomarker measurements. An EBM was fit using the YA and OA groups as control and disease populations, respectively, and a maximum likelihood event sequence was obtained together with sampling uncertainty (Fig. 1A). As this method is Bayesian we did not directly estimate significance, instead a stricter estimate of uncertainty in the maximum likelihood event sequence was estimated by bootstrap resampling of the data and refitting the model 100 times (Fig. 1B).

The resulting event sequence implicates decline in visuospatial associate memory (measured using the CANTAB PAL first trial memory score), hand‐eye coordination (using the NEPSY‐II car/motorbike score), and semantic verbal fluency as early events associated with the likely development of significant AD neuropathology in older adults with DS. Although these tasks cover different cognitive domains, they all rely on sustained attention to perform well, indicating a common underlying ability. Tests of object memory and planning/rule learning such as the Tower of London defined midsequence events, while late events were defined by informant‐rated scales of everyday function and cognitive ability (DLD, short ABS, and revised OMQ; Fig. 1A). Bootstrapping shows a high degree of certainty in the event sequence (Fig. 1B).

### Staging

**Figure 2:**
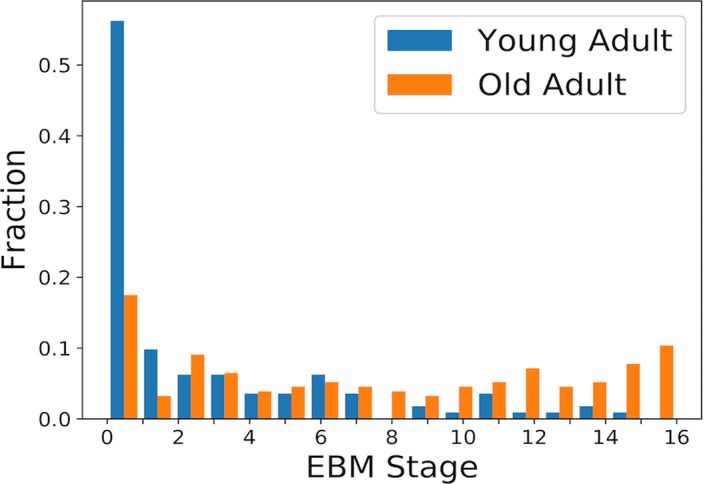
Histogram of event‐based model stages for all participants, colored by age group.

Using the maximum‐likelihood event sequence a disease stage was assigned to each participant. The distribution of stages in the YA and OA groups (Fig. 2) show that the YA group is significantly more likely to be at the earlier stages of the disease (*U* = 4489.5, *p *<* *0.001). The OA group, which was assumed to all have some degree of AD neuropathology, shows a spread along the event stages.

**Figure 3:**
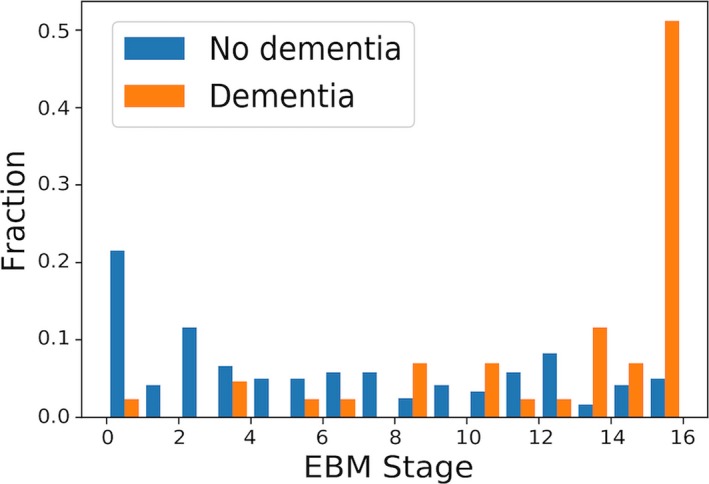
Histogram of event‐based model stages for old adult participants, colored by clinical diagnosis.

As the disease population used in this model (i.e. the OA group) consists of individuals who do not have a clinical diagnosis of dementia, we further analysed the stages of the OA group comparing those with and without clinical dementia diagnoses (Fig. 3). From this we see that individuals with a clinical diagnosis of dementia are significantly more likely to be staged later according the EBM (*U* = 743, *p *<* *0.001).

**Figure 4:**
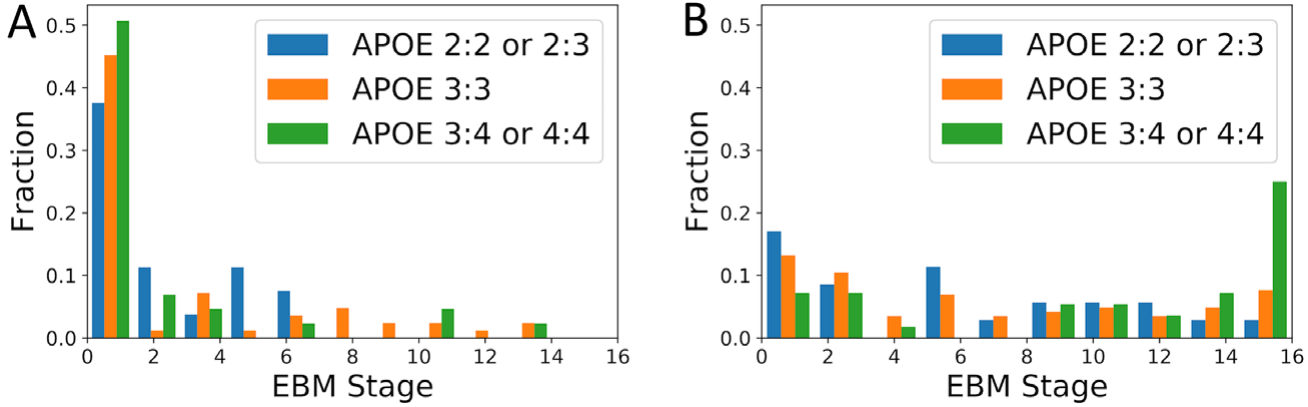
Histogram of event‐based model stages colored by *APOE* genotype. (A) Young adults (B) Old adults.

Splitting individuals based on *APOE* genotype, we saw no relationship with disease stage in the YA group (Fig. 4A), however, in the OA group we observed that individuals in the APOE4 group were significantly more likely to be staged later according to the EBM compared to both the APOE2 (*U* = 205.5, *p *<* *0.002) and APOE3 (*U* = 950, *p *<* *0.001) groups (Fig. 4B).

## Discussion

Using data from nearly 300 adults with DS, we have applied an EBM to characterize the sequence of AD‐related cognitive deterioration in DS. Our estimated event ordering represents the first AD progression model of this type in DS, a population at exceptionally high genetic risk of developing dementia and representing the majority of genetic AD cases. Results suggest decline in visuospatial paired associate memory, hand‐eye coordination, and semantic verbal fluency may be relatively sensitive events during the prodromal stage of AD in DS. Changes in planning abilities and rule learning/shifting may occur slightly later, while changes in informant‐rated behaviors and abilities appeared latest in the model. These results suggest that direct cognitive tests may be more sensitive to early changes than informant‐rated questionnaires. This highlights the need for baseline cognitive assessments in this population to enable early intervention, as subtle changes in cognitive tests may be seen before carers identify decline. The staging distribution of individuals with clinical dementia diagnoses provides validity to the model, and suggests that clinical diagnoses are generally made at a relatively late stage of AD progression in DS. As additional validation of the model, we showed that older individuals with *APOE* 3:4 or 4:4 genotype were more likely to be allocated to later disease stages than those not possessing an *APOE* e4 allele.

As with sporadic AD, in DS there is a time lag of up to several decades between the development of AD neuropathology and meeting the threshold for clinical dementia diagnosis.^2^, ^36^, ^37^ Memory decline, particularly for episodic memory, is viewed as the classic presenting symptom of AD, which gradually progresses to involve other cognitive domains.^38^ However, studies of familial AD mutation carriers have found that some individuals show decline in measures of sustained attention, executive function, language, or behavior several years prior to dementia diagnosis,^39^, ^40^, ^41^ suggesting the sequence of changes we have shown in our population with DS is comparable to that seen in other genetic AD populations.

Previous EBM models have shown that in sporadic AD, changes in cognitive abilities, including memory and attention, follow changes in CSF biomarkers, but occur before brain volumetric changes.^16^, ^42^ The cognitive tests used in these models have tended to combine several cognitive processes. Here, by including tests for more specific cognitive processes as separate biomarkers, we can look more closely at the sequence of decline in populations where obtaining CSF biomarkers, for example, may be challenging.

It has been suggested that executive function decline and behavioral and personality changes may precede memory impairment in dementia development in DS,^43^, ^44^ with some studies reporting that individuals with DS may present with a frontal‐like dementia syndrome in the earliest stages.^43^, ^45^ A recent systematic review of longitudinal DS studies drew similar conclusions,^46^ however, large variability in the follow‐up period and cognitive tasks used prevented a meta‐analysis, and several of the studies included found, like us, that memory and spatial orientation decline seemed to happen first.^47^, ^48^, ^49^

Test sensitivity is a key challenge when assessing baseline cognition and subsequent decline in DS, and may explain these apparently conflicting findings. Some of the studies in the previously mentioned review relied on informant report, which our model suggests may be less sensitive to early change than direct cognitive assessment. Furthermore, Lautarescu and colleagues highlighted that those with a standardized IQ < 40 had very low scores on the memory tasks used, regardless of their dementia status. The CANTAB PAL and object memory tasks used in our battery (but none of the reviewed studies) allowed us to assess visuospatial memory and immediate and delayed recall of everyday items in the majority of our sample, with fewer than 1% of our younger adults aged 16–35 (including many with IQ < 40) at floor on delayed object memory trials,^11^ suggesting that these specific tests are suitable for those with DS and can identify decline in this population. However, for the object memory task, 40% of our younger adults were scoring at ceiling for the delayed object memory trial, suggesting this test may be insufficiently sensitive for measuring ability changes in those with comparatively strong premorbid delayed object memory. Future studies should perhaps increase the number of objects used to improve sensitivity.

The sequence of events revealed by our model has important clinical implications, and suggests that tests of visuospatial associate memory, hand‐eye coordination, and verbal fluency may be particularly useful to track early, subtle cognitive change in middle‐aged individuals with DS in the preclinical and prodromal stages of AD. These tests may all have decline in sustained attention in common, indicating this may be another important aspect of cognition to track changes. The model also suggests that informant‐reported measures may be more useful somewhat later in the course of progression in the lead‐up to dementia diagnosis, and to monitor progression after diagnosis.

AD staging based on EBM could help clinicians track decline during the early stages of cognitive decline in DS, and enable earlier diagnosis that might be beneficial by allowing for timely care‐planning and support. It could also be of use to distinguish a typical sequence of events associated with AD development from the reversible decline that might be due to other, treatable, comorbidities, such as depression or hypothyroidism. Equally promising is the potential use of this model to enable AD staging in clinical trials to select appropriate participants with DS for particular trials, for example those designed to prevent or delay onset of dementia in the prodromal stages. It might also be possible to use such models in the analysis of clinical trial data, by comparing different treatments or placebo controls in terms of progression along the stages of the model. However, we acknowledge that data from further longitudinal studies may be required to confirm and refine the staging model for these applications. Due to the flexibility of the model we can confirm our staging model by using follow‐up data in the model independently, and also by staging patient's follow‐up scores and checking the monotonicity of patients EBM stage trajectories.

This analysis is based on one of the largest and most detailed studies of cognitive decline in DS to date. Participants completed a battery of cognitive tests, which were specifically designed to cover domains commonly affected by DS, including aspects of memory, motor coordination, and executive functions. The tests were adapted to be suitable for use in individuals with DS and selected to minimize reliance on verbal ability, a known area of weakness in individuals with DS. Tests were validated in older adults with DS^50^ before being applied in a large sample including both younger and older adults with DS which allowed for selection of tests with acceptable floor and ceiling effects.^11^ However, we acknowledge the lack of tasks specifically focusing on verbal memory, which is often one of the earliest domains affected in AD.

By using an innovative approach and designating older adults with DS as likely being along a decline trajectory regardless of dementia status, it allowed us to provide a more complete picture of the sequence of events associated with the progression of AD in DS than has been possible to date. Furthermore, the EBM methodology allows for inclusion of individuals who had floored on some tests, which is usually a major limitation to cognitive testing in older individuals with DS.^10^

In conclusion, we have used a data driven approach to overcome some of the common issues in analysis of cognitive data in individuals with DS, and our results reveal that the sequence of events in the progression of AD in DS is comparable to events during the development of AD in other populations, including those with autosomal dominant AD. Specifically, the event sequence suggests that early decline in memory and sustained attention is followed by decline in planning and rule learning/shifting, and occurs before behavioral symptoms as reported by informants. These results help to clarify uncertainties about the sequence of events and staging of AD in DS. Future work including longitudinal data in such models will improve our understanding of decline due to AD in DS further, and will help to improve dementia diagnosis, as well as to inform selection of cognitive outcome measures in future clinical trials to prevent or delay the development of dementia during the prodromal period.

## Conflicts of Interest

The authors declare no conflicts of interest.

## Author Contributions

AS conceived the adult cohort study in conjunction with LonDownS principal investigators. CMS, RH, and SH contributed significantly to recruitment and data collection. KYM and JH contributed to genetic analysis and interpretation of the genetic data. NF, PW, DCA, and AS designed the data analysis. NF analysed the data. NF, CMS, RH, SH, and AS wrote the paper. All authors contributed to the reviewing and editing of the paper.
